# The Effects of Age and the Expression of SPARC on Extracellular Matrix Production by Cardiac Fibroblasts in 3-D Cultures 

**DOI:** 10.1371/journal.pone.0079715

**Published:** 2013-11-06

**Authors:** Jessica Trombetta-eSilva, Erik P. Eadie, Yuhua Zhang, Russell A. Norris, Thomas K. Borg, Amy D. Bradshaw

**Affiliations:** 1 Department of Craniofacial Biology, Medical University of South Carolina, Charleston, South Carolina, United States of America; 2 Department of Regenerative Medicine and Cell Biology, Medical University of South Carolina, Charleston, South Carolina, United States of America; 3 Gazes Cardiac Research Institute, Division of Cardiology, Department of Medicine, Medical University of South Carolina, Charleston, South Carolina, United States of America; 4 Ralph H. Johnson Department of Veteran’s Affairs Medical Center, Charleston, South Carolina, United States of America; Medical University of South Carolina, United States of America

## Abstract

Fibrillar collagen is the primary component of the cardiac interstitial extracellular matrix. This extracellular matrix undergoes dramatic changes from birth to adulthood and then into advanced age. As evidence, fibrillar collagen content was compared in sections from neonates, adult, and old hearts and was found to increase at each respective age. Cardiac fibroblasts are the principle cell type that produce and control fibrillar collagen content. To determine whether fibroblast production, processing, and deposition of collagen differed with age, primary cardiac fibroblasts from neonate, adult, and old mice were isolated and cultured in 3-dimensional (3D) fibrin gels. Fibroblasts from each age aligned in fibrin gels along points of tension and deposited extracellular matrix. By confocal microscopy, wild-type neonate fibroblasts appeared to deposit less collagen into fibrillar structures than fibroblasts from adults. However, by immunoblot analysis, differences in procollagen production and processing of collagen I were not detected in neonate versus adult fibroblasts. In contrast, fibroblasts from old mice demonstrated increased efficiency of procollagen processing coupled with decreased production of total collagen. SPARC is a collagen-binding protein previously shown to affect cardiac collagen deposition. Accordingly, in the absence of SPARC, less collagen appeared to be associated with fibroblasts of each age grown in fibrin gels. In addition, the increased efficiency of procollagen alpha 1(I) processing in old wild-type fibroblasts was not detected in old SPARC-null fibroblasts. Increased levels of fibronectin were detected in wild-type neonate fibroblasts over that of adult and old fibroblasts but not in SPARC-null neonate fibroblasts versus older ages. Immunostaining of SPARC overlapped with that of collagen I but not to that of fibronectin in 3D cultures. Hence, whereas increases in procollagen processing, influenced by SPARC expression, plausibly contribute to increased collagen deposition in old hearts, other cellular mechanisms likely affect differential collagen deposition by neonate fibroblasts.

## Introduction

Fibrillar collagen is the primary component of the interstitial extracellular matrix (ECM) of the heart. The cardiac ECM facilitates alignment of myocytes and maintains mechanical stability of cardiac tissue [1]. Of the ECM components present in the cardiac interstitium, amounts of collagen type I are highest with lesser amounts of collagen III and V represented [2]. These 3 collagen types are the primary components of the collagen fibers in the heart and take on representative structures in the forms of weaves, struts, and coils [3]. Cardiac fibroblasts are considered the primary cell type responsible for synthesis and maintaining homeostasis of the ECM components that comprise the cardiac interstitium [4]. 

As the murine heart develops from neonatal stages to those of adult, the cardiac interstitium undergoes notable changes [5]. Furthermore, the cardiac interstitium continues to undergo changes with increased age. Notably, levels of cardiac fibrillar collagen rise as adults progress to advanced age (>18 months) [6]. Cellular mechanisms that influence amounts of fibrillar collagen in the myocardium include procollagen processing [7]. Collagen I, for example, is synthesized as procollagen with N and C-terminal propeptides that are cleaved prior to incorporation into insoluble ECM [8]. SPARC, a matricellular collagen-binding protein, is one factor shown to influence procollagen processing by cardiac fibroblasts [9]. We therefore sought to determine whether changes in processing in the absence of SPARC expression were apparent in cardiac fibroblasts from neonate, adult and old myocardium. 

Although fibroblasts grown in 2-dimensional (2D) cultures synthesize and secrete ECM proteins, cellular mechanisms that rely on 3-dimensional (3D) environmental factors are not present [10]. For example, assembly of ECM is greatly enhanced in a 3D cellular milieu in which tension can be maintained. Collagen gels provide a suitable 3D culture for some purposes, such as measurements of cell-mediated contraction [11]. However, studies in which cellular mechanisms of collagen production and deposition are under investigation, collagen gels are not ideal due to high levels of exogenous collagen in gels as well as down-regulation of endogenous collagen production by cells in collagen gels. Fibrin gels are a viable alternative 3D substrate for cell culture as cells can be resuspended in fibrinogen and then become embedded in fibrin gels upon cleavage by thrombin [12,13]. In addition, the placement of two pins in a silicone bed beneath the fibrin gels provides points of tension around which fibroblasts will align and contract the fibrin gel to form a tendon-like structure [14]. 

In the current study, cardiac fibroblasts from neonate, adult and old mice were embedded in fibrin gels to determine whether production and processing of fibrillar collagen demonstrated age-specific changes. In addition, production of fibronectin, an ECM protein known to influence collagen I fibril assembly in vitro [15], by fibroblasts at different ages was assessed. Cardiac fibroblasts from wild-type (WT) and SPARC-null mice were evaluated to determine whether differential effects of SPARC expression on ECM production and deposition occurred in fibroblasts from different ages. We report that age-specific and SPARC-dependent changes in ECM production and procollagen processing were found using fibrin 3D cultures. 

## Materials and Methods

### Mice

Mice colonies were maintained at the Medical University of South Carolina (MUSC) animal care facility and all fibroblasts isolations were conducted in strict accordance with the Guide for the Care and Use of Laboratory Animals (National Research Council, National Academy Press, Washington, DC, 1996) and were approved by the Institutional Animal Care and Use Committee at MUSC (Approval ID: ACORP 511). The generation of SPARC-null mice (C57Bl/6) has been described previously [16].

### Cardiac Collagen Histology

Hearts from neonate, adult and old wild-type and SPARC-null mice were removed, rinsed in phosphate-buffered saline (PBS) and fixed in formalin and embedded in paraffin. Seven µm sections were stained with picrosirius red as previously described, imaged under polarized light and quantified using image SigmaScan software [6,17]. Values presented for WT and SPARC-null adult and old mice were published previously [6].

### Primary Fibroblast Culture

For each neonate cardiac fibroblast preparation, six WT and six age-matched SPARC-null neonate (day 1 and day 2 after birth) mice were used. For each primary adult cardiac fibroblast preparation, four 3-4 month WT and four age-matched SPARC-null C57Bl/6 mice were used to isolate cells as reported previously [9]. For each old cardiac fibroblast preparation, four 18-24 months WT and four age-matched SPARC-null C57Bl/6 mice were used to isolate cells. Results of each experiment reported were carried out using four separate primary cell isolations from each age group. Briefly, hearts were excised, dissected free of atria, and minced in PBS containing antibiotics. Tissue was then resuspended in collagenase (Blendzyme 3, Roche, Indianapolis, IN) diluted 1:10 in DMEM (Invitrogen) and incubated at 37° C until tissue was fully digested (1 - 4 hours). Digested tissue was then triturated, resuspended in growth media (DMEM, 10% FCS, with antibiotics and antimycotics) and cells recovered by centrifugation. Cells were rinsed three times in growth media prior to final plating in growth media. All experiments were carried out using cells from passage 2 to 4. 

### 3D Fibroblast Cultures

**Figure 1:**
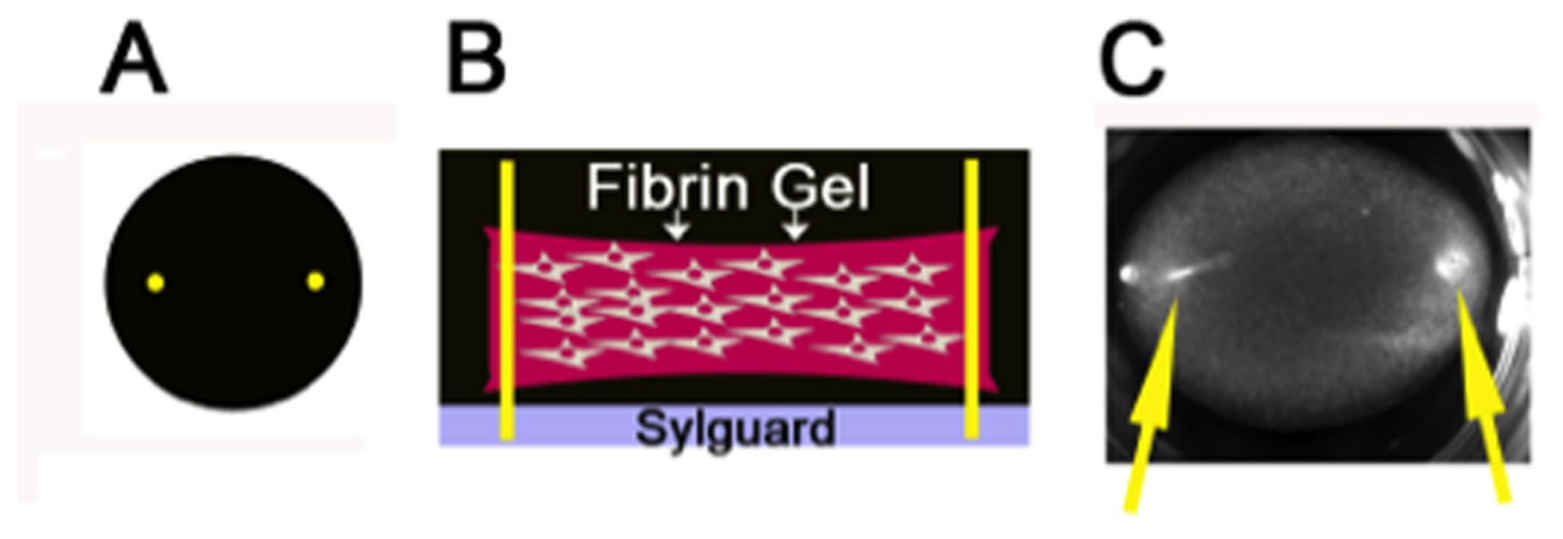
Schematic Diagram of 3-Dimensional Fibrin Gel. A) Schematic top view of 24-well fibrin gel with pins shown in yellow. B). Schematic side-view of fibrin gels in 24-well plate. Fibrin gel containing primary fibroblasts is shown in pink. Yellow insect pins are inserted into a pre-plated layer of sylguard (shown in gray) to provide points of tension that enhance cell alignment and extracellular matrix deposition. C). Actual image of a fibrin gel containing cells cultured in a 24-well plate. Yellow arrows indicate insect pins.

Each well of a 24-well plate was coated with 250 µL of Sylguard (Dow Corning Corporation) and placed at 37 °C overnight. Insect needle pins were inserted perpendicular to the plate 3mm from the well edge (Figure 1). After sterilization by UV light, each well was blocked with 3% bovine serum albumin in PBS for 1 hour to diminish cell attachment to tissue culture plastic. 

Primary cardiac fibroblasts were detached from cell culture plates with trypsin. Three x 10^5^ fibroblasts were resuspended in 748 µL of 5 mg/mL fibrinogen in growth media (DMEM, 5% fetal calf serum (FCS), 5 ng/mL TGF-ß, 50 µM ascorbate, 0.2% primocin). Thrombin (1U/mL) was added to cleave fibrinogen to fibrin and induce gel formation, cells resuspended in fibrinogen/thrombin in growth media (total volume: 750 µl) were transferred to a prepared 24-well plate in which insect pins were previously placed. The gels were incubated at 37 °C for 30 min to allow for polymerization followed by the addition of 500 µl of growth media to each well. Cells in fibrin gels were incubated at 37°C for 18-24 hours at which time conditioned media was collected and fibrin gels were rinsed 3 times for 5 minutes with PBS and then cut in half. Half of the fibrin gel was fixed in either 4% paraformaldehyde (immunofluorescence) or 2.5% glutaraldehyde (transmission electron microscopy, TEM) for 30 min at room temperature, and half of the fibrin gel was stored at -80° C in 100 µl of 2.5% SDS + protease inhibitors (PI, Complete Mini EDTA Free, Roche) for immunoblot analysis. 

### Immunoblotting

Fibrin gels in 2.5% SDS + PI were thawed, tumbled at 4°C for 4 hours, boiled for 5 min, vortexed, boiled for 5 min, and centrifuged at 13.2 RPM for 10 min. Proteins were stored in aliquots at -80 °C. Protein concentration was determined by bicinchoninic acid assay (BCA, Pierce). Equal amounts of protein were separated by SDS-PAGE, transferred to nitrocellulose membranes and probed with appropriate primary and secondary antibodies as described [9]. 

### Confocal and Electron Microscopy

Fibrin gels were fixed in either 2.5% Glutaraldehyde (TEM) or 4% paraformaldehyde for confocal microscopy. Gels were blocked in 2% donkey serum, 0.3% triton PBS (PBS-T) for 1 hour and then incubated with appropriate primary antibodies (anti-murine collagen I (MD Biosciences) and anti-murine SPARC (R&D systems)), diluted 1:200 in 2% donkey serum PBS-T, for 1 hour. Gels were washed 4 times 7 min, and then incubated with appropriate secondary antibody, diluted 1:200 in 2% donkey serum PBS-T (Alexa Fluor, Invitrogen). Gels were washed 4 times 7 min, and then mounted on slides with Prolong Antifade DAPI reagent (Invitrogen). Slides were dried overnight, and were imaged using Olympus Fluoview 1000 confocal laser scanning microscope. Immunofluorescent data were gathered from the full thickness of the fibrin gel and compressed into a single Z-stack image for export. Confocal images shown represent compressed Z-stack images.

### Immunofluorescence Quantification

Quantification of SPARC and collagen I immunolocalization was determined for each individual section in the Z-stack generated from imaging the full thickness of the fibrin gel. The average of the overlap coefficient was presented as the overlap factor (Olympus Fluoview: version 2.1b Software). 

To quantify fibronectin immunofluorescence, the compressed Z-stack images were split into separate RGB files. The ‘R’ image was used to perform standard thresholding of the images, and quantification of positive pixels was determined per image (Image J64). The positive pixels were then normalized to the total image pixel area and averaged from multiple Z-stacks per age and genotype. 

### Statistical Analysis

To determine statistical significance, Student T-test between samples or ANOVA analysis followed by the Bonferonni comparison was performed as indicated for data presented in each figure. Statistical significance was defined as p<0.05. 

## Results

### Levels of Cardiac Fibrillar Collagen Increase with Age in Murine Hearts: Reductions in Levels of Collagen in the Absence of SPARC

**Figure 2:**
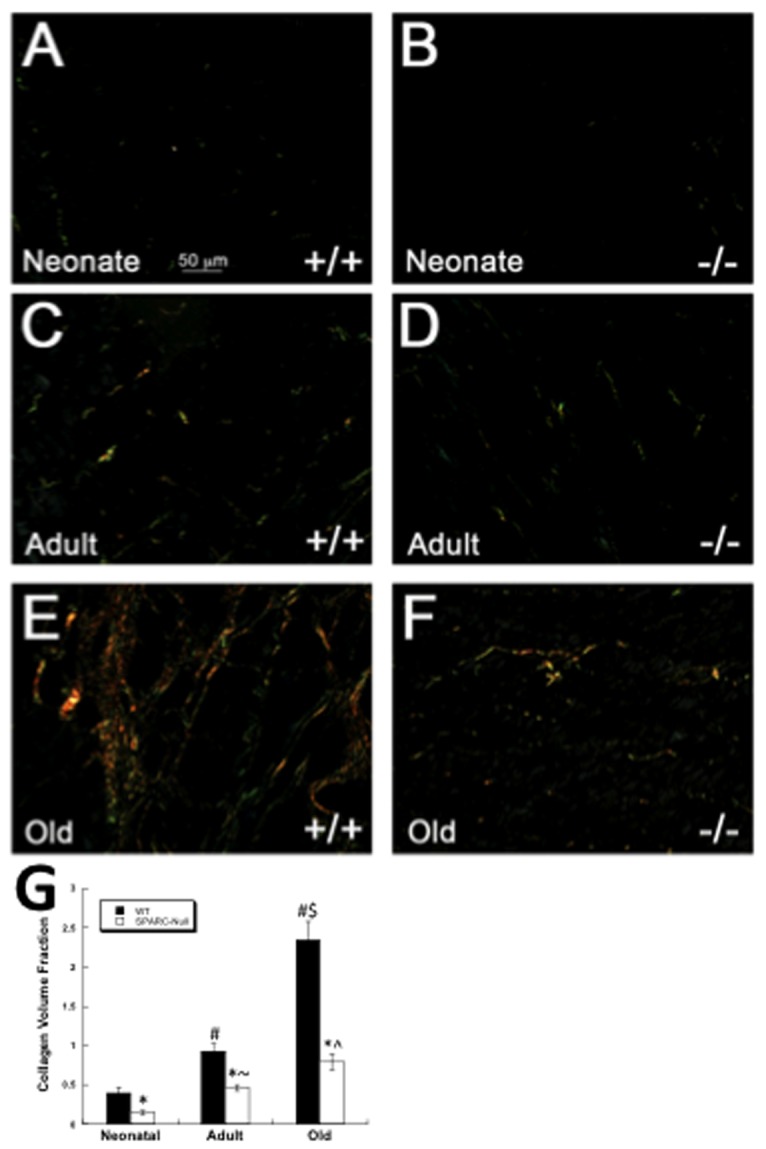
Cardiac Fibrillar Interstitial Collagen Changes with Age. Picrosirius red stained images of cardiac sections viewed under polarized light. Sections from wild-type (WT, +/+) neonates (A) demonstrated diminished fibrillar collagen in comparison to WT adult (C) sections. Increased amounts of fibrillar collagen were apparent in hearts of WT old (E) mice. Cardiac sections taken from SPARC-null neonates (B, -/-), adult (D), and old (F) mice exhibited reduced amounts of PSR stained fibrillar collagen versus age-matched WT mice. Size bar in A = 50 µms; each panel is of equal magnification. G). Quantification of collagen volume fraction from neonate, adult and old hearts. Values presented for WT and SPARC-null adult and old animals were published previously [6]. * p<0.05 versus WT at each age, #p<0.05 vs. WT neonate, $ p<0.05 vs. WT adult, ^ p<0.05 vs. SP-null adult, ~p<0.05 vs. SP-null neonate.

To characterize differences in cardiac fibrillar collagen in neonate (day 1-2), adult (3-4 months), and old (18-24 months) mice, picrosirius red (PSR) staining was performed on sections of hearts from neonate (Figure 2 A and B), adult (C and D), and old (E and F) mice. Section from WT neonate hearts (Figure 2A) demonstrated low levels of fibrillar collagen compared to WT adult mice (Figure 2C). Sections from old WT animals showed increased amounts of PSR-stained collagen versus sections from adult mice. Cardiac sections from SPARC-null (B, D, and F) mice revealed diminished levels of PSR-stained collagen fibers at each age in comparison to age-matched WT mice. Quantification of PSR-stained images, collagen volume fraction, is presented in Figure 1G.

### Production of Collagen In 3D Fibrin Gels by Neonate, Adult, and Old Primary Fibroblasts with and without Expression of SPARC

**Figure 3:**
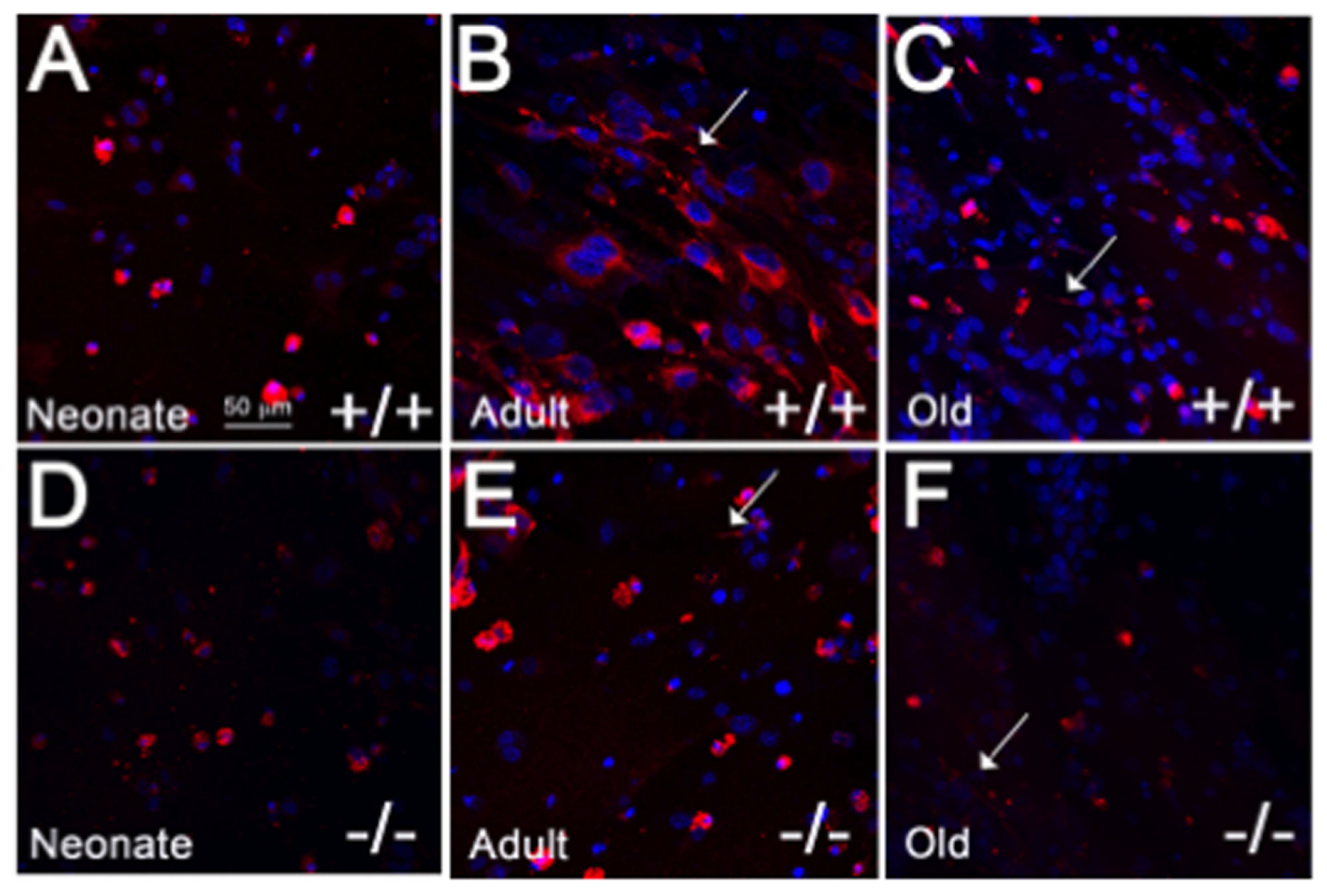
Collagen I Production by Primary Cardiac Fibroblasts in 3D Cultures. Primary cardiac fibroblasts were cultured overnight in 3D fibrin gels with points of tension provided by insect pins. Collagen I immunostaining (red) in WT (+/+) neonates (A) was primarily cell-associated whereas collagen I immunostaining in adult WT fibroblasts (B) showed greater collagen I intensity associated with cells that appeared more aligned and elongated than those in neonate cultures. Extracellular immunostaining associated with fibrillar structures (arrows) were apparent in WT adult and old (C) fibroblast cultures. Primary fibroblasts from SPARC-null (-/-; D, Neonate; E, Adult; F, Old) hearts exhibited lesser collagen I immunostaining in comparison to WT cells. Nuclei are stained blue. Size bar in A = 50 µms; each panel is of equal magnification.

**Figure 4:**
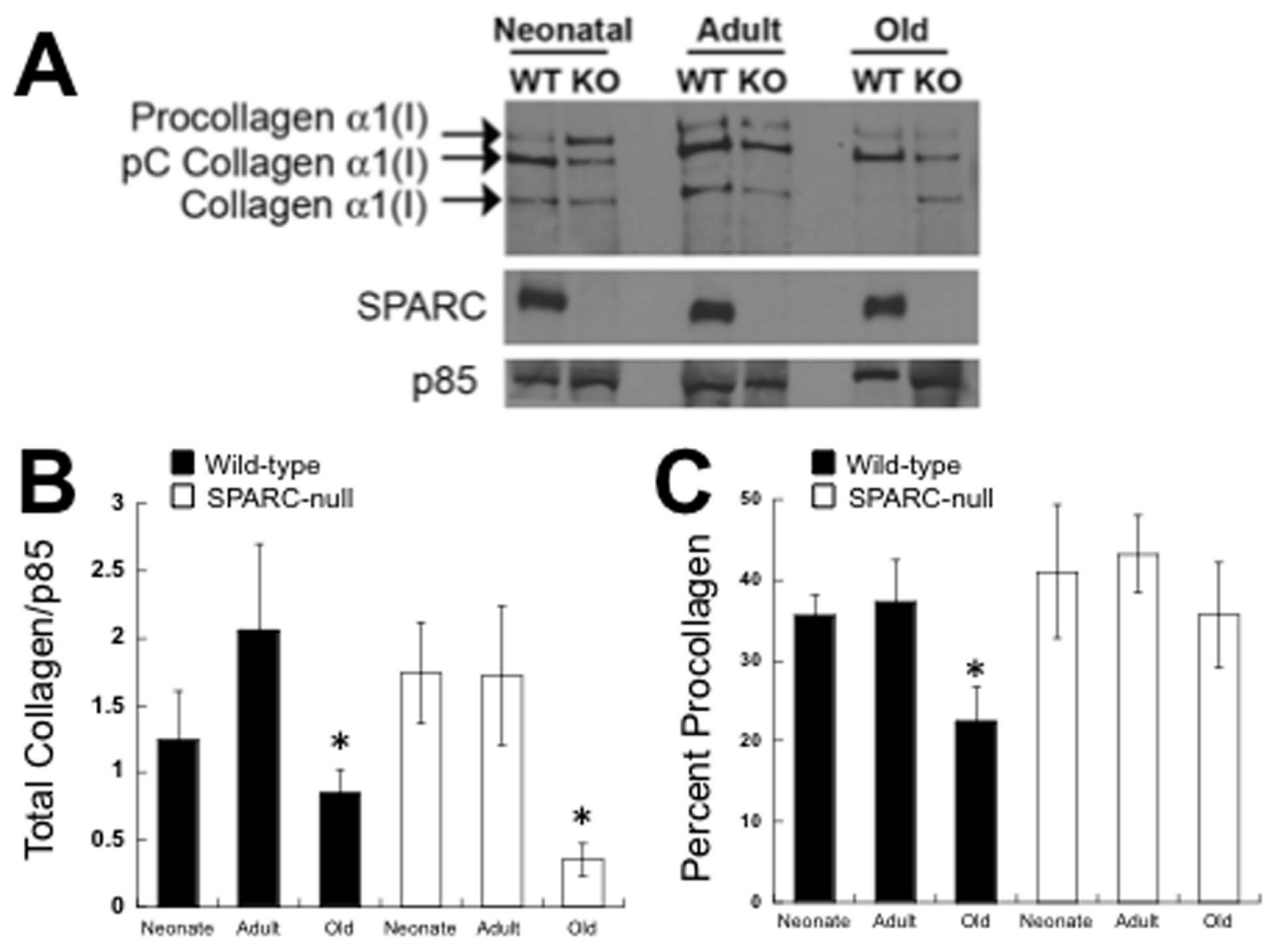
Immunoblot Analysis of Collagen Produced by Cardiac Fibroblasts in Fibrin Gels. A). Procollagen processing and total amounts of soluble collagen recovered from primary fibroblast cultures were performed using immunoblot analysis with anti-murine collagen I antibodies. Three protein bands representing procollagen alpha 1(I), pC collagen alpha 1(I), and fully processed collagen alpha 1(I) were detected on blots. A representative blot is shown in (A). In addition to collagen I, levels of SPARC and p85 (housekeeping control) are shown from each type of primary culture. B). Quantification of collagen I immunoblots (n=4 for each age and genotype) showed significantly less total collagen I (i.e. procollagen, pC collagen, and processed collagen) was detected in fibrin gels populated with old fibroblasts versus neonate or adult cultures. *p<0.05 versus respective genotype-specific neonate and adult values by Student T-test. C). Quantification of collagen I immunoblots (n=4 for each age and genotype) demonstrated that of the collagen I produced by WT old fibroblasts, a significantly decreased proportion of this total collagen was present as procollagen. * p<0.05 versus either neonate or adult WT fibroblasts.

Primary cardiac fibroblasts were isolated from neonate, adult and old mice and cultured in 3D fibrin gels with points of tension provided by insect pins anchored in Sylguard beneath the gels (Figure 1). The 3D environment created by the fibrin and the points of tension supports fibroblast alignment and ECM deposition [14]. Cells in fibrin gels were stained with polyclonal antibodies against murine collagen I (Figure 3). Differences in cellular localization of collagen I immuno-staining were apparent in fibroblasts from different ages. Firstly, the population of neonate cardiac fibroblasts tended to be smaller and appeared less elongated and more rounded in 3D cultures in comparison to adult cardiac fibroblasts. Immuno-staining for collagen I was found associated with cells and appeared to be primarily intracellular in nature although the rounded appearance of neonate fibroblasts increased the intensity of the collagen I staining in cells expressing collagen I (Figure 3A). In cardiac fibroblast cultures from adult and old mice, cells were more elongated and demonstrated greater cell alignment along the plane of tension. Notably, old cardiac fibroblasts appeared smaller than adult counterparts. Accordingly, in contrast to neonate fibroblasts, collagen I immunostaining was visible in distinct cellular locations and indications of insoluble fibrillar collagen deposition were apparent (Figure 3, B and C, arrows). Fibrillar structures that appeared to be predominantly extracellular were visible in individual images from Z-stack confocal images (Figures S1 and S2). In cultures populated by old fibroblasts, collagen I staining was not as robust as that in adult cells (see also Figure 4). 

To determine whether the absence of SPARC affected levels of collagen I in 3D cultures, primary cardiac fibroblasts in 3D from SPARC-null mice were assessed for collagen I immunofluorescence staining. As shown in Figure 3 D-F, the intensity of collagen I immunostaining appeared to be decreased in SPARC-null cardiac fibroblasts from each age in comparison to WT cells. In general, patterns of expression between neonate, adult, and old fibroblasts were similar in SPARC-null versus WT cells in that adult fibroblasts demonstrated the greatest amounts of collagen I immunostaining versus neonate and old cultures. 

### Quantfication of Soluble Collagen I and Procollagen Processing by Cardiac Fibroblasts from Different Ages

Further biochemical characterization of collagen production by fibroblast cultured in 3D gels was carried out by immunoblot analysis to determine total levels of soluble collagen I produced and differences in procollagen I processing by fibroblasts from hearts of different ages. Whereas immunoreactivity of collagen I in fibrin gels detects both soluble and insoluble fibrillar collagen, immunoblot analysis detects only soluble collagen in the form of procollagen alpha 1(I), pC collagen alpha 1(I) and collagen alpha 1(I) that is not covalently cross-linked into insoluble collagen fibers. Collagen alpha 1(I) that is incorporated into insoluble ECM in the form of collagen fibers is not soluble in extraction buffer. Figure 4A shows an immunoblot probed with anti-collagen alpha 1(I) antibodies representing the forms of collagen I produced by cardiac fibroblasts that were detected in fibrin gels populated with cardiac fibroblasts (Figure 4A). Total amounts of soluble collagen alpha 1(I) (i.e. procollagen I + pC collagen I + collagen I) produced by WT and SPARC-null cells was quantified using independent cell preparations from neonate, adult, and old mice (Figure 4B). Similar levels of total soluble collagen were associated with fibroblast cultures from neonate and adult animals whereas significantly less soluble total collagen I was detected in gels populated with old fibroblasts. A similar trend was demonstrated by SPARC-null fibroblasts from these ages. 

In 3D fibrin gels, procollagen alpha 1(I) was processed by fibroblasts from each age to yield the intermediate pC collagen alpha 1(I), and collagen alpha 1(I) (Figure 4 A and C). Measurement of procollagen processing was determined by calculating the percent of total collagen represented as procollagen. The percent of unprocessed procollagen significantly decreased in old WT cultures in comparison to that of neonate or adult cells. Thus, although old fibroblasts demonstrated diminished levels of total soluble collagen in 3D fibrin gels versus neonate and adult cells, the procollagen produced underwent more efficient procollagen processing to yield pC collagen alpha 1(I). The reduced levels of soluble collagen alpha 1(I) in old WT cultures might result from increased incorporation of this processed collagen alpha 1(I) to insoluble ECM or a reduction in processing of the C-propeptide of pC collagen alpha 1(I). In contrast, SPARC-null fibroblasts did not demonstrate significant differences in procollagen processing in old fibroblasts in comparison to neonate and adult cells. As shown in Figure 4A, levels of SPARC in fibrin gels populated by WT fibroblasts from different ages were not significantly different. 

### Overlap of SPARC and Collagen I Immunoreactivity in 3D Cultures

**Figure 5:**
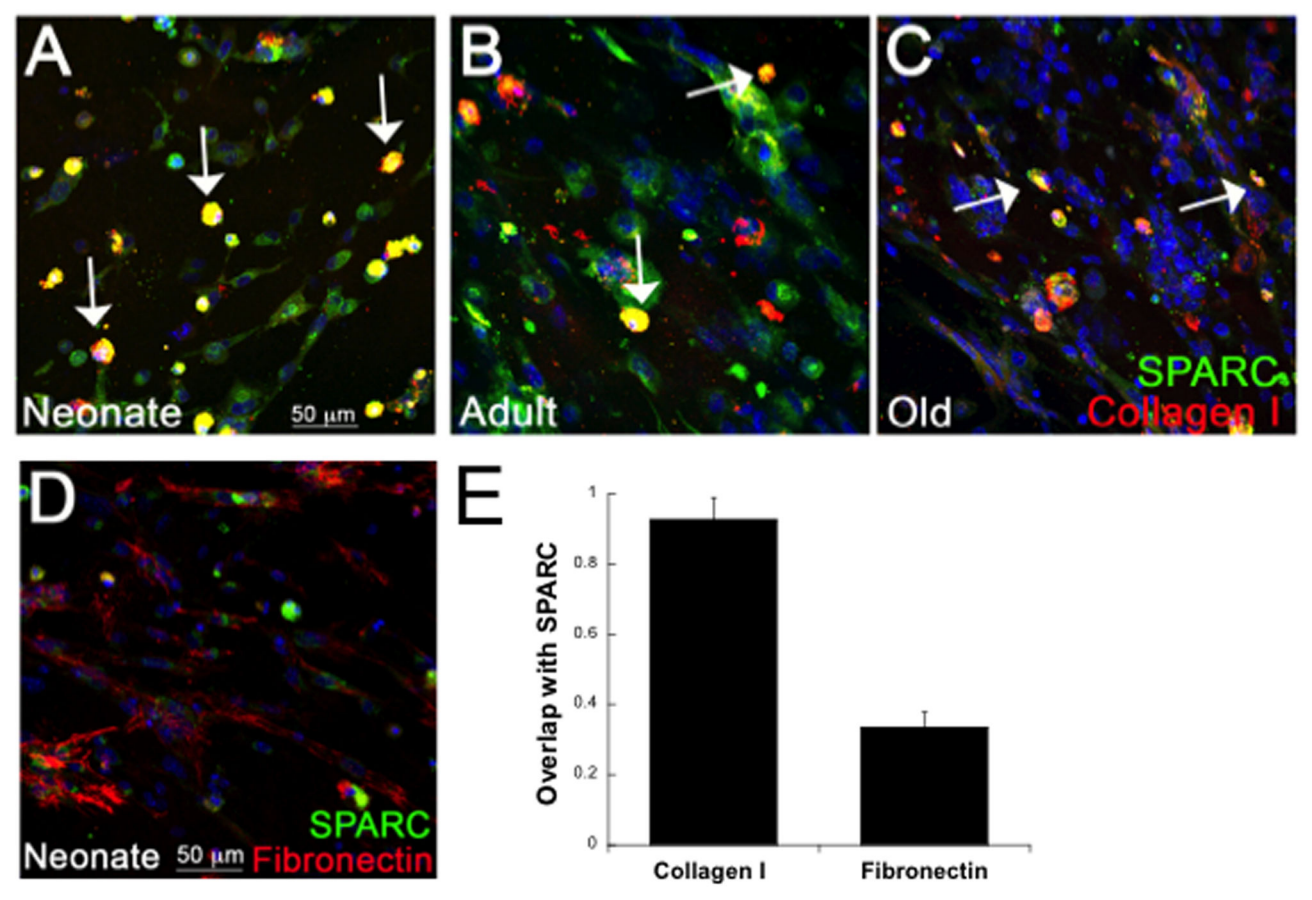
Overlap of Collagen I and SPARC Immunoreactivity in 3D Cardiac Cultures. Cardiac fibroblasts from WT neonates (A), adult (B), and old (C) hearts cultured in fibrin gels were co-stained with antibodies against collagen I (red) and SPARC (green). Sites at which collagen I and SPARC immunoreactivity overlapped is shown in yellow (arrows). D). Fibronectin and SPARC staining in neonate cultures showed little incidence of overlap. E). Coincident staining was quantified using the overlap coefficient (n=4 separate primary cell preparations, See Methods) in cultures of neonate fibroblasts co-stained with SPARC or either collagen I or fibronectin. Size bar in A = 50 µms; each panel is of equal magnification.

To determine whether staining of SPARC, a characterized collagen-binding protein, overlapped with that of collagen I, cardiac fibroblasts in fibrin gels were stained with antibodies against collagen I and SPARC. As shown in Figure 5 A-C, significant overlap of SPARC and collagen I was found in WT cultures. Coincidence of staining (yellow) of SPARC (green) and collagen I (red) was strongest in neonate fibroblasts in which staining for both proteins appeared to be predominantly intracellular or cell-surface associated (arrows; Figure 5A and E). To this point, the majority of cells in neonate cultures appeared round and less elongated than older cells in culture. Nonetheless, the majority of neonate cells expressing collagen I were also positive for SPARC staining. In cultures from adult and old hearts, immunostaining of collagen I was associated with both cellular and fibrillar structures predicted to be extracellular. Similar to neonate cultures, coincident staining of SPARC and collagen I was found primarily with the portion of collagen I that was cell-associated (arrows). Hence, in adult and old cultures, a lower incidence of overlap in co-staining for collagen I and SPARC was found (Figure 5, B and C). Previously, SPARC was not found to bind to fibronectin (Fn) *in vitro* [18]. Consistent with this result, no significant overlap in Fn and SPARC-staining was found in 3D fibrin cultures (Figure 5, E).

### Differential Production of Fibronectin by Cardiac Fibroblasts from Different Ages

**Figure 6:**
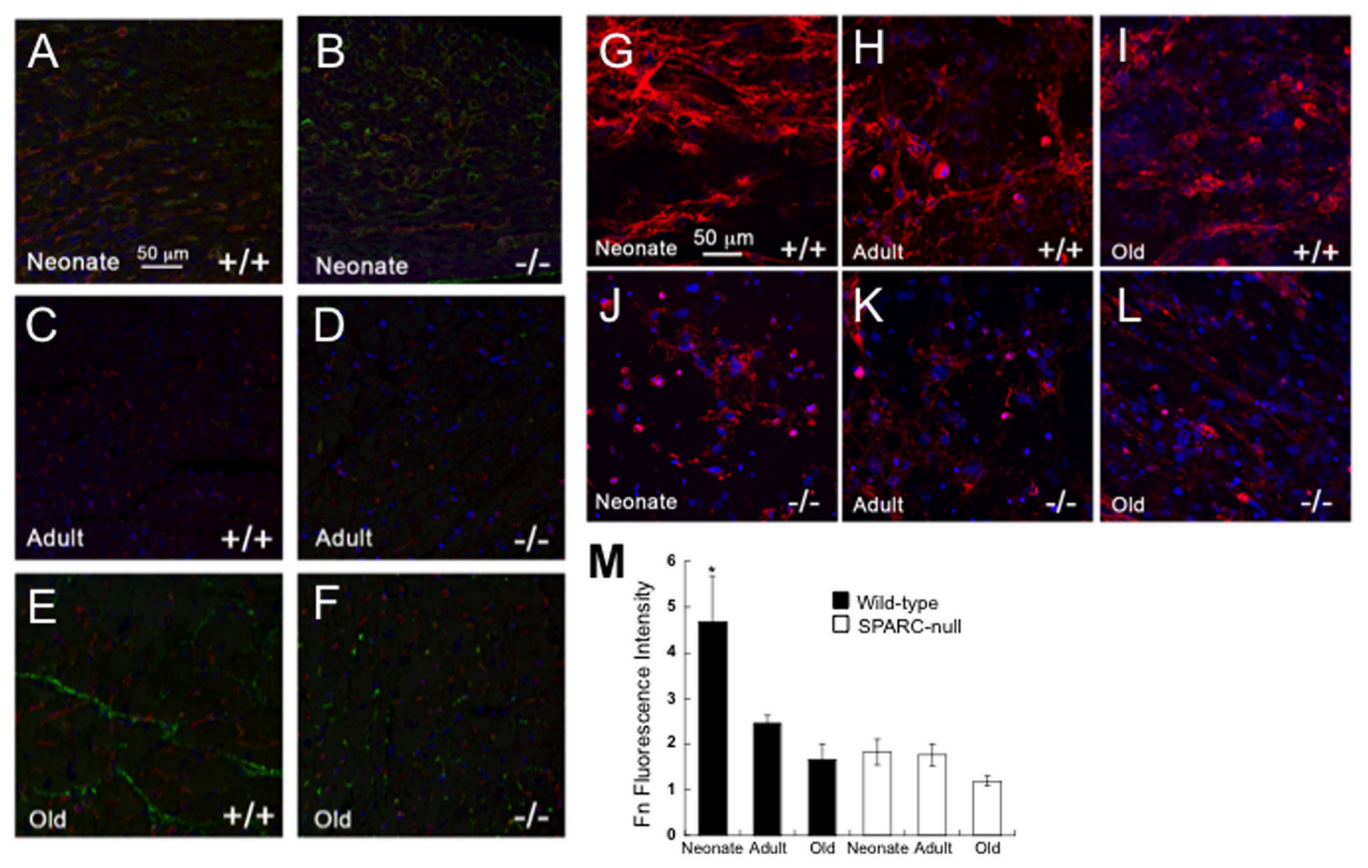
Levels of Fibronectin Immunoreactivity in Heart Sections and Fibronectin Production by Primary Cardiac Fibroblasts is Influenced by Age. A-F). Fibronectin immunoreactivity in sections of heart from WT (+/+) neonate (A), WT adult (C), WT old (E), SPARC-null (-/-) neonate (B), SPARC-null adult (D), and SPARC-null old (F) mice. G-L). Fibronectin immunoreactivity in fibrin gels populated with WT (+/+) neonate (G), WT adult (H), WT old (I), SPARC-null (-/-) neonate (J), SPARC-null adult (K), or SPARC-null old (L) fibroblasts. Size bar in A = 50 µms; each panel is of equal magnification. M). Quantification of fluorescent intensity for fibronectin immunostain in fibrin gels. WT neonate fibroblasts demonstrated significantly increased levels of fibronectin immunoreactivity versus the other conditions. * p< 0.02 by one-way ANOVA analysis.

To test whether immunostaining of Fn differed with age, antibodies generated against Fn were used to probe cardiac sections from neonate, adult and old hearts (Figure 6, A-F). Neonate sections from WT mice displayed higher levels of immunostaining for Fn in comparison to adult mice. In sections from old WT hearts, some cardiac regions showed increased Fn localization in comparison to those of adult WT hearts. Fn immunostaining at each age did not co-localize with a marker of endothelial cells (not shown). Thus Fn staining was primarily associated with interstitial fibroblasts rather than vascular structures. 

In WT fibroblast cultures grown in 3D, the highest intensity of Fn staining was observed in neonate cardiac fibroblast cultures in which Fn immunolocalization was associated with a fibrillar network (Figure 6, G-L). Fn-positive structures were also found in cardiac fibroblast cultures deposited by adult and old cells, although the intensity of Fn staining was not as great as that of the neonates. In the absence of SPARC, significantly less Fn was associated with SPARC-null neonate fibroblasts versus cells from WT neonates (Figure 6, M). In addition, the intensity of Fn staining did not appear to change in fibroblasts from different ages in the absence of SPARC expression, in contrast to collagen I staining (Figure 3). 

### Ultra-Structural Analysis of Cardiac Fibroblasts From Different Ages in 3D

**Figure 7:**
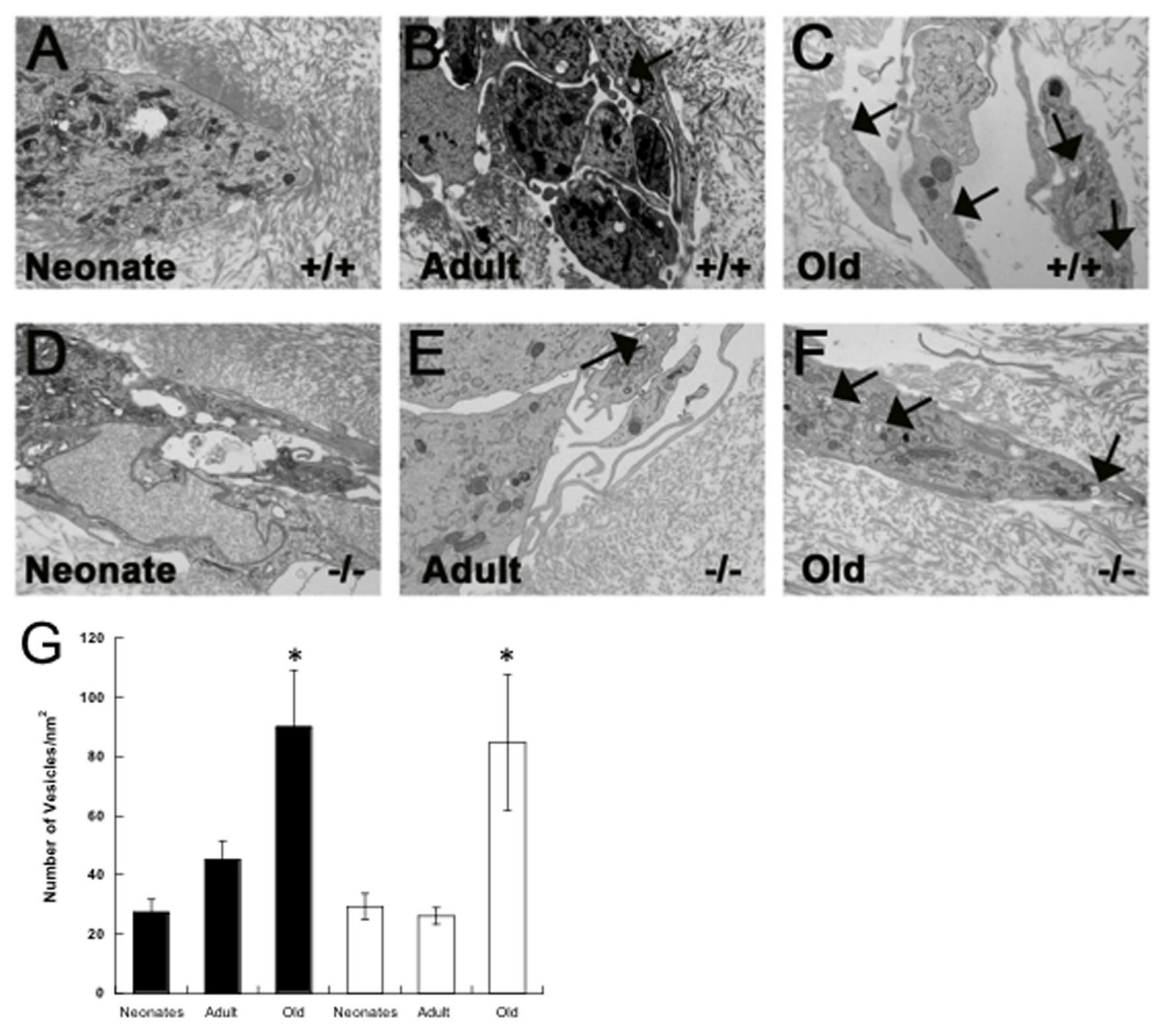
Ultrastructural Analysis of Primary Fibroblast from Different Ages in Fibrin Gels. A-F) Electron microscopy (EM) was performed on fibrin gels populated with WT (+/+) neonate (A), WT adult (B), WT old (C), SPARC-null (-/-) neonate (D), SPARC-null adult (E), and SPARC-null old (F) fibroblasts. Arrows indicate vesicles containing structures with a round profile that resemble collagen fibrils. G). Quantification of vesicles visible by EM that contained structures resembling round collagen fibrils. A higher incidence of these structures was found in cells from old mice of each genotype. *p< 0.05 versus neonate or adult fibroblasts by Student T-test.

Ultra-structural analysis of cardiac fibroblasts in fibrin gels was performed by transmission electron microscopy (EM). As shown in Figure 7, neonate, adult, and old fibroblasts grown in fibrin gels exhibited vesicles reminiscent to those observed in EM images of embryonic tendon and periodontal ligament. Vesicles in these tissues have been postulated to represent sites of collagen fibril assembly and deposition [19,20]. Similar vesicles observed in 3D fibrin cultures contained round profiles consistent with collagen-fibril containing vesicles (Figure 7 A-F, arrows). Interestingly, quantification of the number of vesicles per area of individual cell showed a higher number of vesicles in fibroblasts from old animals versus those of neonate and adult origin (Figure 7G). 

## Discussion

The cardiac ECM demonstrates age-dependent changes in levels of fibrillar collagen (Figure 2). We sought to determine whether primary cardiac fibroblasts isolated from mice of different ages exhibited variations in collagen production and procollagen processing that might contribute to age-dependent changes in the cardiac interstitium. Primary fibroblasts were cultured in 3D fibrin gels with points of tension created by insect pins to promote cell alignment and ECM deposition (Figure 1, [14]). Our results showed that, although primary fibroblasts from the hearts of neonates produced and processed procollagen I in 3D fibrin gels, these cells did not appear to align or assemble collagen into extracellular fibrillar structures to the same extent as fibroblasts from adult or old hearts. Clearly, sections of neonate hearts demonstrated reduced amounts of fibrillar collagen as well. In contrast, fibroblasts from old mice produced lower amounts of collagen, as indicated by immunofluorescence and immunoblot analysis. Interestingly, the collagen produced by old fibroblasts underwent more efficient processing of procollagen I to pC collagen I. However, as represented in Figure 4A, levels of soluble fully processed collagen alpha 1(I) were not elevated in old cultures. At least two possible mechanisms might explain these results: 1) reduced amounts of soluble collagen alpha 1(I) reflect increased incorporation of collagen alpha 1(I) to an insoluble ECM, 2) decreased levels of C-proteinase activity in old cultures reduce processing of pC collagen alpha 1(I). Whereas enhanced processing of procollagen by cardiac fibroblasts might be one mechanism that contributed to increased collagen accumulation in the hearts of old mice, reduced amounts of ECM collagen incorporation in neonate hearts did not appear to depend on differences in procollagen processing. 

Ultrastructural analysis of primary fibroblasts grown in 3D fibrin gels revealed the presence of intracellular vesicles containing fibril-shaped cargo. The vesicles were reminiscent of those described in embryonic tendon and periodontal ligament that have been proposed to act as collagen fibril assembly organelles, or “fibripositors” [19,20]. Whether the vesicles observed in these primary cardiac fibroblast cultures contained collagen fibrils was not determined, however this fibrin gel system has been used to culture tendon fibroblast where similar structures were also observed [14]. Interestingly, cells from old mice contained significantly higher numbers of these vesicles per cell than younger counterparts (Figure 7). If in fact these vesicles are reflective of greater collagen fibril forming capacity coupled with the knowledge that old cells also demonstrated increased procollagen processing, then cardiac fibroblasts from old animals might be more efficient in collagen fibril assembly and deposition. This increased capacity might contribute to increased collagen accumulation in old hearts. 

Although sections of hearts from neonates did not show abundant fibrillar collagen by PSR staining, immunostaining of Fn, an ECM protein implicated in collagen fibril assembly in vitro, was evident in neonate hearts [15]. The intensity of Fn staining also appeared to be greater in the hearts of neonates in comparison to that of adult mice (Figure 6). Similarly, Fn immunostaining was more robust in 3D cultures populated with neonate cardiac fibroblasts versus those populated with fibroblasts from adult or old animals. As Fn has been proposed to serve as a scaffold for collagen fiber assembly in fibroblast cultures, perhaps neonate cardiac fibroblasts produced higher levels of Fn that precedes collagen fiber deposition. Interestingly, increased Fn staining was also found in old hearts associated with sites of interstitial collagen deposition (Figure 6). In the absence of SPARC, Fn levels were lower in neonate cells consistent with previous results found in SPARC-null lung fibroblasts where decreased Fn assembly in vitro was observed [21].

SPARC is a collagen-binding matricellular protein shown to be required for fibrosis in a number of different tissues including heart [22]. Normal hearts of SPARC-null adult and old mice also show reduced levels of fibrillar collagen at baseline ([6], Figure 2). We report herein that neonate SPARC-null hearts likewise demonstrated diminished amounts of fibrillar collagen. Hence, cardiac fibroblasts from WT and SPARC-null mice at each age point were analyzed for their capacity to process, deposit, and assemble collagen in 3D gels. Immunostaining for collagen I in 3D fibrin gels revealed decreased intensity of staining in SPARC-null fibroblasts from neonates, adults, and old hearts (Figure 3). However, immunoblot analysis of total collagen from WT versus SPARC-null cultures did not reveal significant differences in amounts of collagen produced with the exception of old fibroblasts. Similarly, differences in procollagen processing were detected only in comparing processing by old fibroblasts from WT versus SPARC-null hearts. Increases in procollagen processing shown for old WT fibroblasts were not found in old SPARC-null fibroblasts. Hence the decrease in collagen accumulation noted in old SPARC-null hearts might be influenced by decreased efficiency of procollagen processing by SPARC-null fibroblasts. 

The lack of differences in procollagen processing in neonate and adult cardiac fibroblasts in the absence of SPARC expression suggested that additional cellular mechanisms influence SPARC-dependent collagen deposition by cardiac fibroblasts at these ages. In 2-dimensional cultures, adult SPARC-null cardiac fibroblasts exhibited greater cellular association of collagen I as well as collagen V. Cell-associated, overlap of SPARC and collagen I immunostaining detected by confocal microscopy was most pronounced in neonate cultures (Figure 5). In fibroblast cultures from adult and old mice, immunostaining of collagen I was found associated with fibrillar structures that resembled assembled collagen and tended not to demonstrate significant overlap with SPARC immunoreactivity to the same degree as collagen on cells. We conclude that SPARC bound to collagen is preferentially associated with newly secreted procollagen and influences procollagen interaction with cell surfaces [9]. In this scenario, once procollagen is processed and perhaps further modified appropriately, SPARC disengages and collagen I is efficiently incorporated into insoluble collagen fibrils. 

In conclusion, these studies have shown that primary cardiac fibroblasts isolated from neonates, adults, and old mice demonstrated significant differences in collagen processing, deposition and assembly. The use of a 3D fibrin gel with points of tension facilitated the analysis of procollagen production and processing by cardiac fibroblasts in a milieu that more closely resembles that of tissue. Future experiments using this system will promote the discovery of cellular mechanisms governing collagen fibril deposition and assembly in the cardiac interstitium. 

## Supporting Information

Figure S1 **Individual images from a Z-stack imaging fibronectin immunoreactivity in WT neonate cultures.** Arrows designate fibronectin fibrils that appear extracellular, arrowheads designate nuclei. Numbers designate sequential captured images.
(PPTX)

Figure S2 **Individual images from a Z-stack imaging collagen I immunoreactivity in SP-null old cultures.** Arrows designate collagen fibrils that appear extracellular, arrowheads designate nuclei. Numbers designate sequential captured images.
(PPTX)
