# Supplementary figures and images for: The Effects of Age and the Expression of SPARC on Extracellular Matrix Production by Cardiac Fibroblasts in 3-D Cultures
Source: PLoS One. 2013 Nov 6;8(11):e79715. doi: 10.1371/journal.pone.0079715 (PMC3819255; doi:10.1371/journal.pone.0079715)

## Slide 1
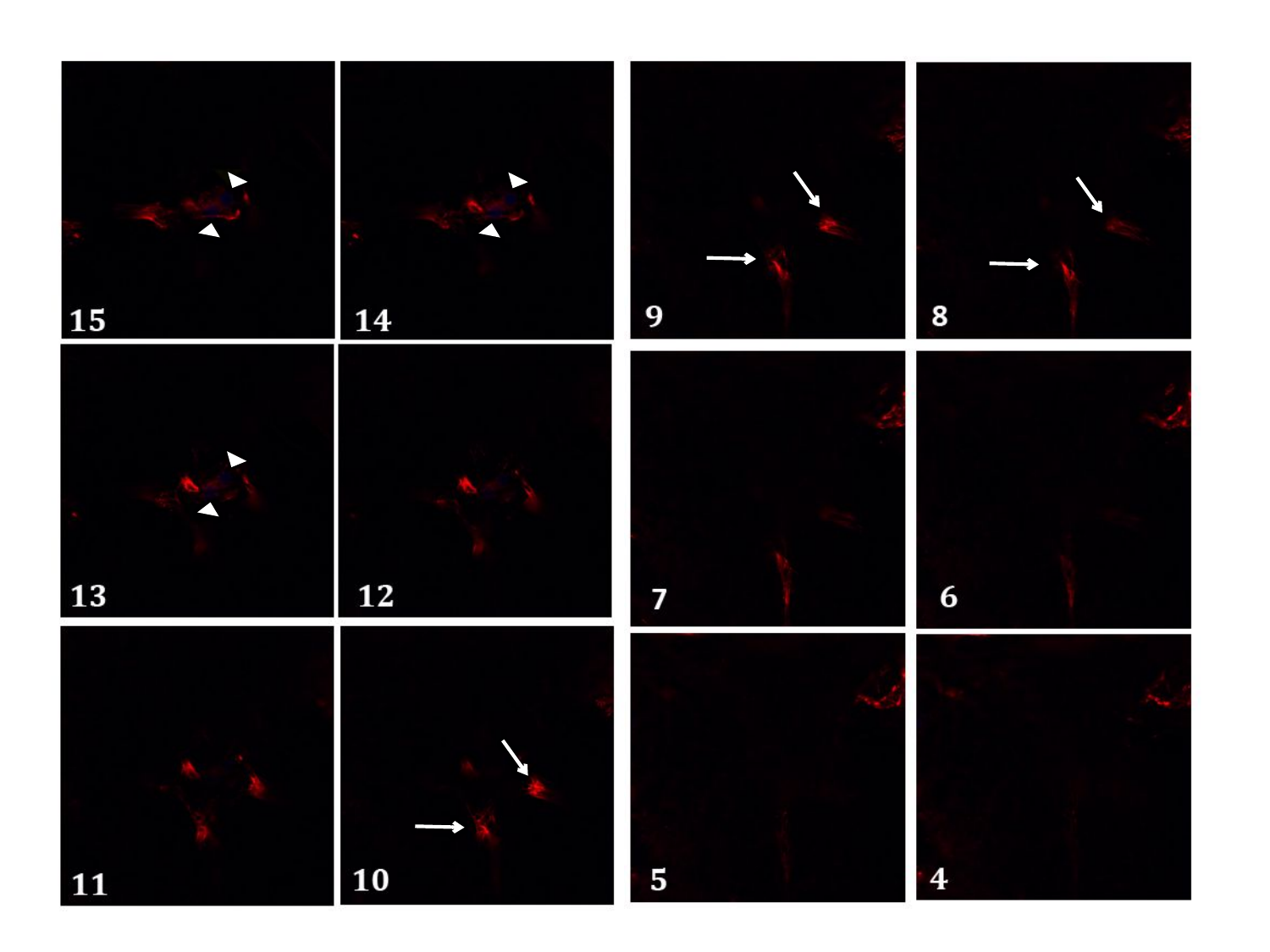

Supplement: Figure S1 — Individual images from a Z-stack imaging fibronectin immunoreactivity in WT neonate cultures. Arrows designate fibronectin fibrils that appear extracellular, arrowheads designate nuclei. Numbers designate sequential captured images. (PPTX) [file pone.0079715.s001.pptx]

## Slide 1
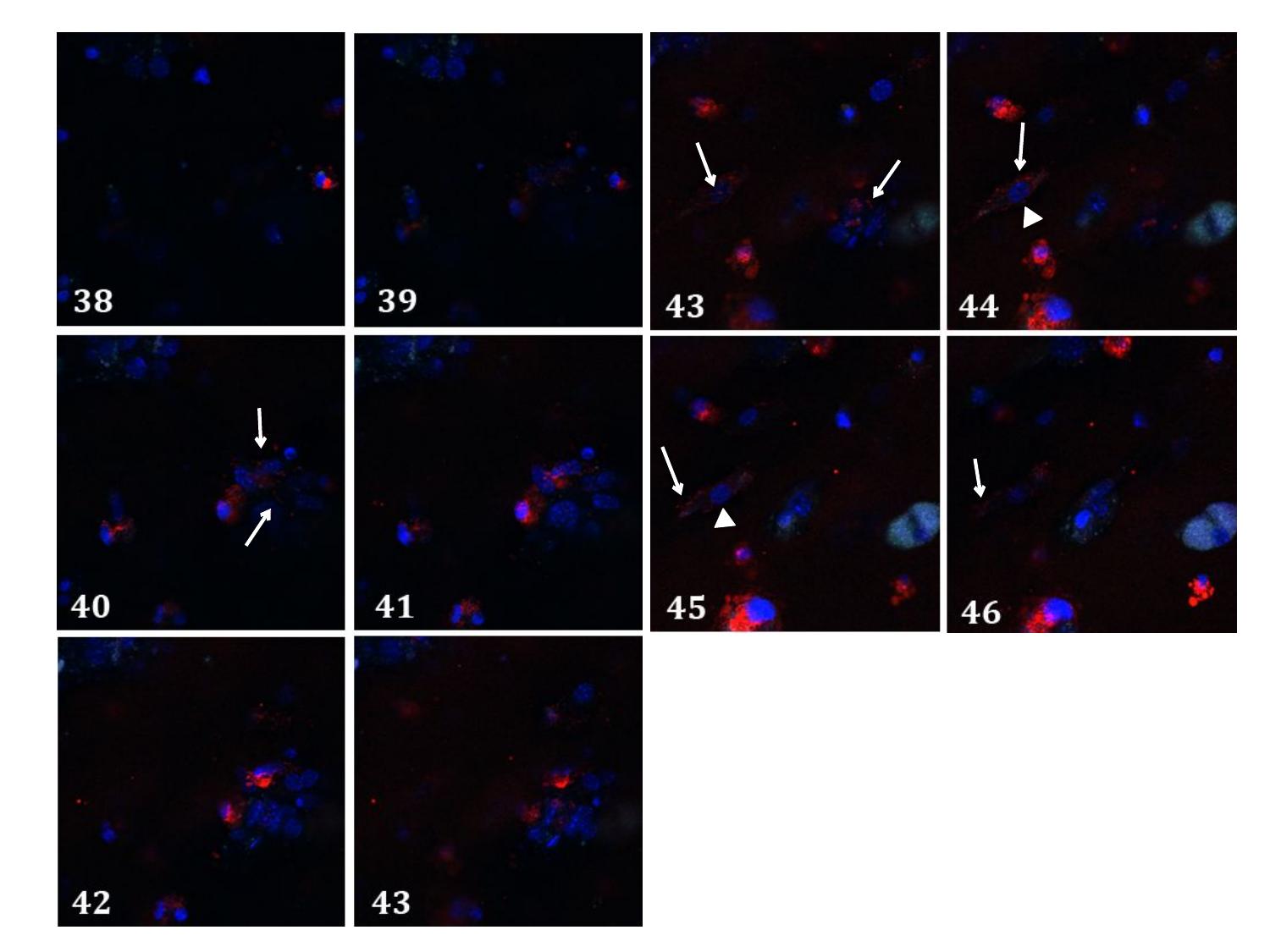

Supplement: Figure S2 — Individual images from a Z-stack imaging collagen I immunoreactivity in SP-null old cultures. Arrows designate collagen fibrils that appear extracellular, arrowheads designate nuclei. Numbers designate sequential captured images. (PPTX) [file pone.0079715.s002.pptx]
